# Perineal body–sparing transsphincteric anorectoplasty (TSARP) for rectovestibular fistula: Mid-term functional outcomes from a single center

**DOI:** 10.1007/s00383-026-06485-5

**Published:** 2026-06-15

**Authors:** Berat Dilek Demirel, Başak Dağdemir Ezber, Beytullah Yağız

**Affiliations:** Department of Pediatric Surgery, Ondokuz Mayıs University, Samsun, Turkey

**Keywords:** Anorectal malformation, Rectovestibular fistula, Transsphincteric anorectoplasty, Sphincter sparing

## Abstract

**Purpose:**

This study aimed to evaluate the mid-term functional and cosmetic outcomes of perineal body–sparing trans-sphincteric anorectoplasty (TSARP) in patients with anal atresia and rectovestibular fistula.

**Methods:**

Patients with anal atresia and rectovestibular fistula who underwent perineal body–sparing TSARP between 2018 and 2024 were retrospectively reviewed. All procedures were performed by a single pediatric surgeon. Demographic data, presence of colostomy, perioperative complications, postoperative first-feeding time, and functional outcomes were recorded. Functional outcomes in patients were assessed using the Krickenbeck classification**.**

**Results:**

Twenty-six patients underwent perineal body–sparing TSARP. One-stage repair was performed in 16 patients (61.5%), whereas 10 (38.5%) had a protective colostomy. The median age at operation was 40 days (range 2 days–7 months). Posterior vaginal wall injury occurred in two patients and was repaired uneventfully. Two patients developed wound infection that resolved with local wound care. The mean follow-up duration was 3.25 years. Among 18 patients older than 3 years old, voluntary bowel control was present in 15 patients (83.3%). Constipation occurred in seven patients, and no patient developed soiling.

**Conclusion:**

Perineal body–sparing TSARP appears to be a safe and effective technique for rectovestibular fistula, providing low complication rates with satisfactory functional and cosmetic outcomes.

## Introduction

Anorectal malformations (ARMs) are congenital birth defects that mostly depend on surgical correction and occur in approximately 1 in 4000–5000 live births, encompassing a wide clinical spectrum. In female patients, the most common type is anal atresia with a co-existing rectovestibular fistula (RVF) [1]. The timing and surgical approach for RVF repair may vary depending on the surgeon’s experience and preference, and patients may be treated either in the neonatal period with one-stage repair or later, after creation of a protective colostomy [2, 3]. Several surgical techniques have been described for the treatment of RVF, including posterior sagittal anorectoplasty (PSARP), anterior sagittal anorectoplasty (ASARP), and their modifications. Numerous studies have compared the functional outcomes of these techniques [4, 5]. However, division of the sphincteric complex, disruption of the perineal body, and wound site complications (particularly in patients undergoing one-stage repair) have been associated with poor cosmetic results and functional impairment [6]. For these reasons, alternative surgical approaches aimed at preserving the sphincteric complex and perineal structures have been developed. Rectovestibular fistula is considered the most favorable type of ARM in terms of continence potential, and satisfactory functional outcomes can be expected in the majority of patients when appropriate surgical technique and postoperative management are employed [1, 3].

The primary goals of ARM surgery are to create a neoanus positioned anatomically within the sphincter complex, preserve the structures responsible for continence, and reconstruct the perineal anatomy as close to normal as possible. Preservation of the sphincter complex and perineal structures has been related with improved functional outcomes [7, 8]. Perineal body–sparing trans-sphincteric anorectoplasty (TSARP) is a technique that allows mobilization of the rectum without dividing the sphincter complex while preserving the perineal body [9]. Although transsphincteric approaches to rectovestibular fistula have been previously described in the literature, variations exist regarding surgical technique, perioperative management, and the emphasis placed on perineal body preservation [8–10]. The aim of this study is to present our surgical technique and perioperative management protocol and to evaluate the mid-term functional and cosmetic outcomes of perineal body–sparing TSARP in patients with rectovestibular fistula.

## Materials and methods

### Study design

This retrospective single-center study included patients with rectovestibular fistula who underwent perineal body–sparing trans-sphincteric anorectoplasty (TSARP) between 2018 and 2024. All procedures were performed by a single pediatric surgeon. The study was approved by the Institutional Clinical Research Ethics Committee (approval number: 2025/450).

### Patients

Medical charts of the patients were reviewed for patient demographics, age at operation, presence of colostomy before definitive surgery, intraoperative and postoperative complications, postoperative calibration and dilatation protocol, continence, cosmetic outcomes, and follow-up data.

### Surgical technique

**Fig. 1:**
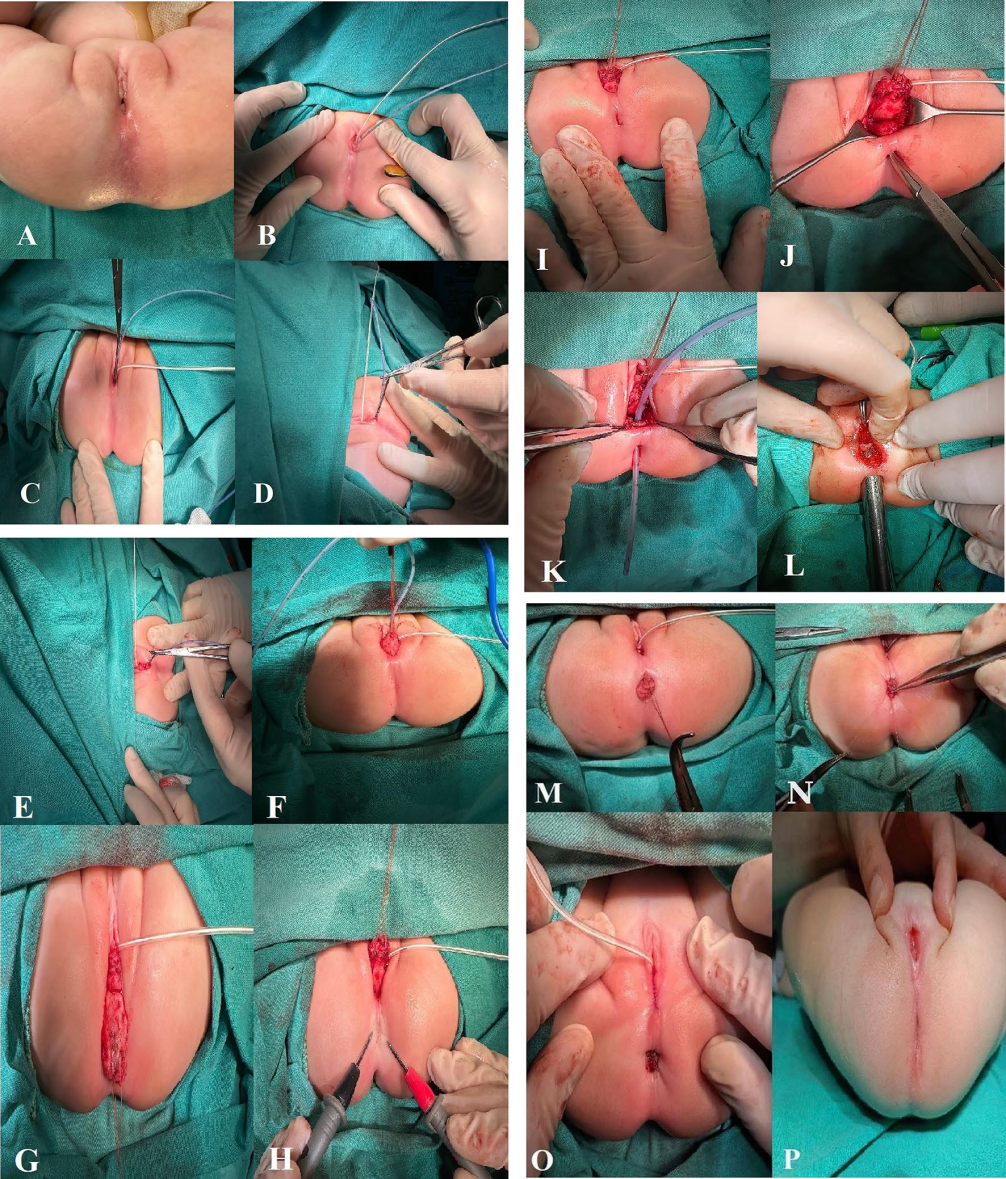
Intraoperative steps of perineal body–sparing transsphincteric anorectoplasty (TSARP) for rectovestibular fistula. **A.** Preoperative appearance of rectovestibular fistula. **B.** Placement of traction sutures around the fistula opening. **C**, **D.** Circumferential incision and initiation of rectal mobilization. **E**–**G.** Dissection of the rectum from posterior vaginal wall. **H**, **I.** Identification of the sphincter complex using electrical stimulation. **J**, **K.** Creation and dilation of the trans-sphincteric tunnel. **L**–**M.** Pull-through of the rectum and completion of recto-anal anostomosis. **N.** Immediate postoperative appearance. **O**, **P.** Postoperative appearance at 1-year follow-up

All procedures were performed under general anesthesia with the patient in the lithotomy position. In patients without colostomy, rectal irrigation with warm saline was performed through a catheter inserted via the fistula to achieve bowel preparation. A Foley catheter was inserted for bladder decompression and to prevent urine contamination of the surgical field. The center of the external sphincter complex was identified using an electrical muscle stimulator, and loupe magnification was used during all stages of the procedure. Traction sutures (6/0 silk) were placed circumferentially at the mucocutaneous junction of the fistula. A circumferential incision was made around the fistula opening, and rectal mobilization was initiated. The rectum was carefully separated from the posterior vaginal wall using sharp and blunt dissection; electrocautery use was minimized and carefully controlled in the rectovaginal plane to reduce the risk of injury to the vaginal wall. When the correct avascular dissection plane between the rectum and the vaginal wall is entered, bleeding is inherently minimal. When minor bleeding was encountered low-power bipolar cautery was used with caution to minimize thermal spread. Adequacy of rectum mobilization was confirmed by two criteria: first, the rectum could be delivered to the level of the neoanus without tension; second, complete separation from the posterior vaginal wall was verified by confirming that traction applied to the rectum did not tent or displace the vaginal wall, followed by direct inspection to ensure the integrity of the vaginal wall. After adequate mobilization of the rectum, the sphincter complex was identified using electrical stimulation. A small midline incision was made at the neoanal site. A tunnel was created through the sphincteric complex using a right-angle clamp under electrical stimulation guidance and gradually dilated using Hegar dilators. The rectum was pulled through the sphincteric complex reaching to the neoanus. Recto-anal anastomosis was completed using absorbable sutures (6/0 vicryl). After confirming the integrity of the vaginal wall, the vestibular defect was closed in layers using absorbable sutures. Special attention was paid to preserve the perineal body and the surrounding sphincter complex during rectal mobilization and pull-through. The rectum was positioned at the center of the sphincter complex under direct vision with the guidance of electrical stimulator. The operative steps are illustrated in Fig. 1.

### Postoperative management

Intravenous cefazolin was administered 30 minutes before surgery and at the 6th postoperative hour for antibiotic prophylaxis. In patients without colostomy, metronidazole was included in the perioperative antibiotic regimen. Oral cephalosporin therapy was continued for 7 days postoperatively. Feeding was initiated at postoperative hour 3 in patients with colostomy. In patients who underwent one-stage repair, oral feeding was initiated on postoperative day 3 to reduce stool contamination of the wound. Local wound care consisted of irrigation with normal saline, drying with sterile gauze, and application of povidone–iodine for local antisepsis. The Foley catheter was removed on postoperative day 5. Rectal calibration was initiated on postoperative day 14. Parents were instructed to perform dilatation twice daily starting on postoperative day 21. The target anal caliber was determined according to age-appropriate reference values as described by Peña et al.[11].

### Follow-up and outcome assessment

Patients were followed weekly during the dilatation period. Follow-up visits were scheduled monthly during the first year, every two months until 2 years of age, and every 6 months thereafter. Dietary counseling was provided at each follow-up visit. Parents were instructed to encourage footstool-assisted defecation posture training, in which the child’s feet are supported during defecation to approximate a squatting position, thereby optimizing the anorectal angle and facilitating evacuation. Functional outcomes were evaluated according to the Krickenbeck classification [12]. Cosmetic outcomes were assessed based on perineal appearance, position of the neoanus, and preservation of the perineal body, and were rated as excellent, good, or poor by both the surgeon and the family at follow-up visits.

### Statistical analysis

Statistical analysis was performed using SPSS software® (version 25, IBM Corp., Armonk, NY, USA). Continuous variables were expressed as median (range) or mean ± standard deviation, while categorical variables were presented as frequencies and percentages.

## Results

### Demographic and operative data

A total of 26 patients who underwent perineal body–sparing TSARP were included in the study. The median age at operation was 40 days (range 2 days–7 months). One-stage repair was performed in 16 patients (61.5%), with a median age at operation of 30 days (range 2–180 days). The remaining 10 patients (38.5%) had a protective colostomy and underwent definitive repair using the same procedure. The median age at operation in these patients was 71.5 days (range 40–210 days).

### Intraoperative and postoperative complications

Intraoperatively, posterior vaginal wall injury occurred in two patients (7.6%), one with a prior colostomy and one undergoing one-stage repair. The vaginal defects were repaired primarily using absorbable sutures (6/0 Vicryl), and no postoperative complications were observed. In the one-stage repair group, two patients developed wound dehiscence on postoperative days 5 and 12. Both patients were successfully managed with local wound care. However, in one patient, a diverting colostomy was performed due to wound dehiscence with concern for progressive wound breakdown. Colostomy closure was performed after complete wound healing, with an uneventful postoperative course; no anal stenosis was identified during subsequent follow-up. No patient required anoplasty revision. Mild mucosal prolapse developed in one patient during the first postoperative year and was successfully treated with mucosal excision. Demographics and surgical outcomes are summarized in Table 1.

**Table 1:** Demographic characteristics and surgical outcomes of patients

Parameter	Patients *n* (%)	One-stage repair *n* (%)	Prior colostomy *n* (%)
Number of patients	26 (100)	16 (61.5)	10 (38.5)
Median age at operation days [range]	40 [2–210]	30 [2–180]	71.5 [40–210]
Time to postoperative feeding	–	3 days	3 hours
Intraoperative complications			
Vaginal wall injury	2 (7.6)	1	1
Postoperative complications			
Wound infection	2 (7.6)	2	0
Mild mucosal prolapse	1 (3.8)	1	0

Values are presented as median (range) or number (%)

### Postoperative follow-up and outcomes

Rectal calibration using Hegar dilators was initiated at postoperative week 2. The median neoanal caliber at the first evaluation was 8 mm (range 7–10 mm). A daily dilation program was initiated from postoperative week 3. After achieving the age-appropriate anal caliber, dilation was continued for an additional eight weeks with gradual reduction. The median duration of the calibration program was 15 weeks (range 12–20 weeks). During follow-up, mild anal stenosis was observed in one patient 3 months after completion of the dilation program and resolved after an additional 8 weeks of dilation.

**Fig. 2:**
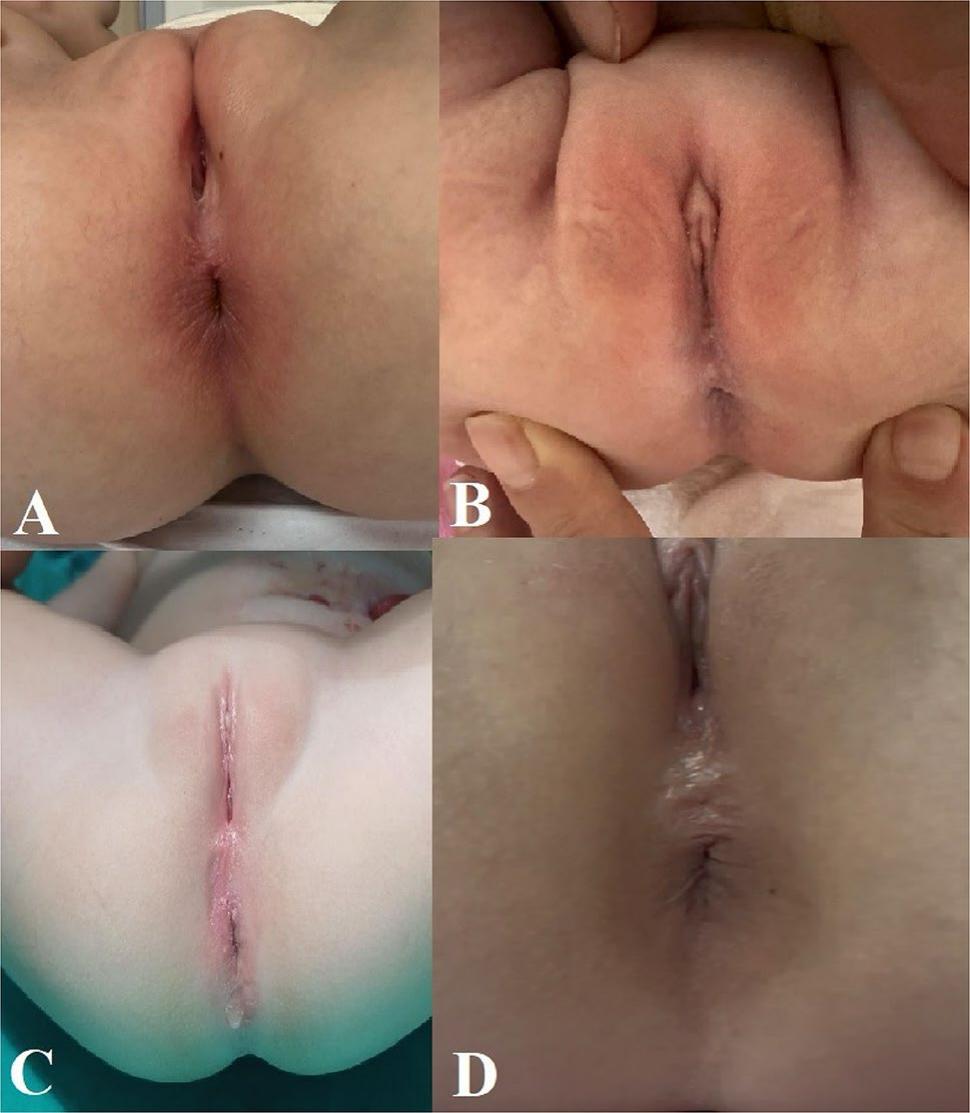
Late postoperative perineal appearance after perineal body–sparing transsphincteric anorectoplasty (TSARP)

The median follow-up period was 3.25 years (range 7 months–8 years). Constipation developed in seven patients (26.9%). According to the Krickenbeck classification, four patients had grade 1 and three had grade 2 constipation, all of whom improved by dietary modification and laxative therapy. No patient had grade 3 constipation. Functional outcomes were evaluated in 18 patients older than 3 years. Voluntary bowel control was present in 15 patients (83.3%) according to the Krickenbeck criteria (feeling of urge, ability to verbalize, and ability to hold bowel movements). Among the remaining three patients, one was 3 years and 2 months old at evaluation, and the other two had co-existing neurological impairment. No patient developed soiling. Mid-term functional outcomes are presented in Table 2. Cosmetic appearance was rated as excellent or good by both the surgeon and the families in all 26 patients; no patient received a poor rating from either assessor (Fig. 2).

**Table 2:** Mid-term functional outcomes after perineal body–sparing TSARP

Parameter	*n* (%)
Follow-up period (years). median (range)	3.25 (0.6–8)
Constipation (total)	7 (26.9)
Krickenbeck grade 1	4 (15.4)
Krickenbeck grade 2	3 (11.5)
Krickenbeck grade 3	0 (0)
Voluntary bowel control^a^	15/18 (83.3)
Soiling	0 (0)
Late anal stenosis	1 (3.8)
Mucosal prolapse	1 (3.8)
Satisfactory cosmetic appearance	26 (100)

^a^Voluntary bowel control was evaluated only in patients aged ≥ 3 years according to the Krickenbeck classification (*n* = 18)

## Discussion

The primary goal of surgical treatment in anorectal malformations (ARM) is not only to establish an anatomically adequate pathway for intestinal evacuation but also to achieve optimal long-term functional and cosmetic outcomes. Rectovestibular fistula (RVF) represents the most common type of anorectal malformation in female patients, and the optimal surgical approach remains a subject of ongoing debate [1, 3]. In addition to anatomical reconstruction, preservation of the sphincter complex and perineal structures plays a critical role in achieving satisfactory continence, functional outcomes, and perineal aesthetics, which are essential for the physical and psychosocial well-being of affected individuals [3, 8, 9].

Posterior sagittal anorectoplasty (PSARP) has long been accepted as the standard surgical procedure for ARM repair and has been widely used with high success rates in large patient series [4, 13]. In contrast, the perineal body–sparing TSARP technique preserves the parasagittal muscle fibers and the sphincter complex, which may contribute to improved functional outcomes and reduced complication rates. Furthermore, the absence of an additional perineal incision may reduce postoperative analgesic requirements and decrease wound-related complications, particularly in patients undergoing one-stage repair. Performing the procedure in the supine position rather than the prone position may also be advantageous from an anesthetic perspective.

Anterior sagittal anorectoplasty (ASARP) and its modifications are also widely used for the treatment of RVF; however, these techniques have been criticized due to the requirement for extensive anterior dissection and the associated risk of vaginal injury [5–7]. Vaginal complication rates following ASARP have been reported to range between 5 and 15% in the literature [5, 6]. In our series, vaginal injury rate of 7.6% is consistent with the range reported in the literature and is not claimed to be a technique-specific advantage; rather, it reflects careful surgical dissection, the use of loupe magnification, and minimized and controlled use of electrocautery in the rectovaginal plane. Early recognition of vaginal injury is critical, and careful verification of vaginal integrity should always be performed. During rectovaginal dissection, electrocautery use should be minimized and carefully controlled; when required, cutting current for dissection and low-power coagulation for hemostasis is preferable to minimize thermal spread to the vaginal wall, as thermal injury may obscure tissue planes through tissue shrinkage and fibrosis. The use of loupe magnification during all stages of the procedure may further facilitate meticulous dissection and improve surgical outcomes. A key advantage of the perineal body–sparing TSARP technique is the preservation of the sphincter complex while allowing controlled transsphincteric passage of the rectum [8, 9].

Recently, Nguyen Phung et al. reported a large single-center series of 67 patients undergoing one-stage trans-sphincter anorectoplasty for rectovestibular fistula [9]. They reported vaginal injury in 6% of cases, a recurrence rate of 1.5%, and voluntary bowel control in 97% of patients older than 3 years, with soiling observed in 5.9% of patients. In comparison, our series demonstrated similar intraoperative complication rates and no cases of soiling, although constipation was observed in a subset of patients. The relatively lower constipation rate in our series may reflect our structured follow-up protocol, which includes dietary counseling and the encouragement of footstool-assisted defecation posture training—a technique that optimizes the anorectal angle and facilitates evacuation. Nevertheless, as constipation is a well-recognized long-term sequela of ARM repair and may develop or worsen over time, the mid-term nature of our follow-up should be considered a limitation, and continued surveillance is warranted.

Compared with ASARP and its modifications, the most important advantage of our approach (particularly in one-stage cases) is the preservation of the perineal body, which may contribute to improved wound healing and fewer postoperative wound complications. Similar to the modified ASSSARP technique described by Elbatarny et al., our approach preserves the external sphincter complex. However, in contrast to ASSSARP, our technique does not require any skin incision over the perineal body; instead, the rectum is routed through a transsphincteric tunnel, leaving the perineal skin and underlying fibromuscular tissue intact [10]. Preservation of the perineal body may not only improve cosmetic outcomes but may also prevent potential gynecological problems such as dyspareunia in adulthood and possible complications during vaginal delivery. We believe that structural preservation of the perineal body may allow spontaneous vaginal delivery in adulthood; however, long-term gynecological follow-up is warranted to confirm this.

In patients with ARM, the congenital absence of a normal anal canal and the hypoplasia of the sphincter complex and parasagittal muscle fibers may impair anorectal sensation and bowel control [3]. When the sphincter complex is preserved and the rectum is positioned within the muscle complex, rectal distension may be perceived more effectively, which may contribute to improved continence. Abdelmohsen et al. compared TSARP, ASARP, modified ASARP, and PSARP in patients with RVF and reported that TSARP provided the best functional and cosmetic outcomes among these techniques [5].

Traditionally, staged surgery with an initial diverting colostomy has been recommended to reduce wound complications and perioperative morbidity. However, recent studies have shown that one-stage repair may be associated with acceptable rates of wound complications [2, 3]. In our practice, one-stage repair is the preferred approach for newborns. Nevertheless, some patients in our series were referred from other centers after colostomy creation, and both approaches appeared feasible within our technique. Although postoperative feeding may be delayed in one-stage patients, prolonged fasting or parenteral nutrition is usually unnecessary, and wound complications were mild and manageable without additional surgical intervention.

Most parents encounter these conditions for the first time when their child is diagnosed with ARM. Therefore, regardless of the surgical technique used, the perioperative course should be carefully explained to families. Proper wound care, hygiene, and adherence to the postoperative dilatation program are essential components of postoperative management. Anoplasty dehiscence occurred in one patient on postoperative day 12, necessitating a diverting colostomy. Although wound dehiscence following anoplasty is multifactorial in nature—potentially involving wound tension, local infection, inadequate tissue perfusion, and suboptimal wound care—structured and repeated parental education regarding wound hygiene may help reduce this risk. In previously reported TSARP series, complication rates have generally been low and functional outcomes satisfactory [9, 13, 14]. Similarly, in our series, no major complication requiring anoplasty revision occurred.

The optimal timing of surgery in RVF remains controversial. While some authors advocate delayed repair following prolonged fistula dilatation to facilitate rectovaginal separation, others favor early repair [5, 9]. In our practice, surgery is performed promptly after diagnosis in hemodynamically stable neonates without significant associated anomalies and prolonged fistula dilatation is not routinely used. Early repair avoids the morbidity of a protective colostomy, enables prompt initiation of the postoperative dilatation program, and allows the fistula to serve as a natural access point for intraoperative bowel preparation. Neonatal tissues are notably pliable, facilitating dissection, and the lower toxic fecal flora during this period may reduce wound contamination risk; furthermore, single-stage anorectoplasty in neonates has been shown to be associated with significantly shorter operative times, fewer postoperative wound complications, and comparable long-term defecation function compared to delayed repair in infants [15]. Additionally, early functional use of the anatomically reconstructed neoanus may contribute to improved sensory development. Nevertheless, patient selection remains paramount, and the timing of surgery should be individualized based on the clinical condition of the patient.

Preoperative preparation for one-stage repair has traditionally included several days of fasting and repeated rectal washouts to minimize postoperative wound complications [5]. However, Lai et al. reported that early postoperative feeding was not associated with increased wound complications [16]. Because the patients in our series were operated on during early infancy and were exclusively breast-fed, prolonged preoperative fasting or extensive rectal washouts were not applied. Instead, intraoperative irrigation performed at the beginning of the procedure was considered sufficient. Our wound complication rates were not higher than those reported in studies using prolonged bowel preparation [5, 7].

Functional outcomes remain the most important indicators of success following ARM surgery. In our series, no patients had grade 3 constipation or soiling according to the Krickenbeck classification. Voluntary bowel control was present in 83.3% of patients older than 3 years, which is comparable to or even favorable when compared with outcomes reported after PSARP and ASARP. Previous studies have suggested that preservation of the sphincter complex may reduce rates of soiling and severe constipation, and our findings are consistent with these observations [5, 7–10, 13, 14].

Cosmetic outcomes represent another important parameter, particularly in female patients and in terms of parental satisfaction. With the perineal body–sparing approach, satisfactory perineal appearance was achieved in all patients, and the integrity of the perineal body was preserved.

Laparoscopic-assisted anorectoplasty (LAARP) has been described as an alternative approach that mobilizes the rectum under laparoscopic visualization, potentially minimizing perineal dissection [17]. Comparative studies have demonstrated the advantages of LAARP particularly in intermediate-type RVF, where the high rectal pouch, long fistula tract, and close proximity to the vagina increase the risk of wound complications with conventional perineal approaches [18]. For rectovestibular fistula, perineal approaches have demonstrated satisfactory outcomes without the need for abdominal access and pelvic dissection [5]. According to Bischoff et al., no justification for laparoscopy could be found in the management of rectovestibular fistula, and in some cases it may even be contraindicated [19]. We therefore believe that a transfistular perineal approach, by avoiding pelvic dissection altogether while preserving the perineal body, may represent a less invasive and equally effective alternative in this specific subgroup. TSARP may therefore be considered a viable surgical option for the treatment of rectovestibular fistula.

This study has several limitations, including its retrospective design, relatively small sample size, and the absence of a control group. In addition, functional evaluation could not be performed in some patients due to their young age. Functional outcomes were assessed using the Krickenbeck classification, which represents the most widely accepted standardized framework for reporting continence outcomes in ARM patients. However, this classification has limitations, including its simplicity and reliance on subjective parental and clinical reporting. Objective anorectal manometry was not routinely performed in our cohort, and future studies incorporating objective physiological assessments would provide a more comprehensive evaluation of functional results. Cosmetic outcomes were assessed using a simple three-tier rating scale (excellent/good/poor) by both the surgeon and the family, rather than a validated scoring instrument, which represents a further limitation of the current study. However, the homogeneous patient group, standardized surgical technique, structured follow-up protocol, and mid-term follow-up period represent important strengths of the study. Furthermore, all procedures were performed by a single surgeon, ensuring technical consistency.

## Conclusion

Perineal body–sparing TSARP appears to be a safe and effective surgical technique for the treatment of rectovestibular fistula in anorectal malformations. Preservation of the sphincter complex and perineal anatomy may contribute to low complication rates, satisfactory continence outcomes, and the absence of soiling. The feasibility of performing the procedure as a one-stage repair represents an additional advantage. Further studies with larger patient populations and longer follow-up are required to confirm the validity and reproducibility of this technique.

## Data Availability

No datasets were generated or analysed during the current study.
